# Extracellular Electron Transfer Is a Bottleneck in the Microbiologically Influenced Corrosion of C1018 Carbon Steel by the Biofilm of Sulfate-Reducing Bacterium *Desulfovibrio vulgaris*

**DOI:** 10.1371/journal.pone.0136183

**Published:** 2015-08-26

**Authors:** Huabing Li, Dake Xu, Yingchao Li, Hao Feng, Zhiyong Liu, Xiaogang Li, Tingyue Gu, Ke Yang

**Affiliations:** 1 School of Materials and Metallurgy, Northeast University, Shenyang, Liaoning, China; 2 Institute of Metal Research, Chinese Academy of Sciences, Shenyang, Liaoning, China; 3 Department of Chemical and Biomolecular Engineering, Institute for Corrosion and Multiphase Technology, Ohio University, Athens, Ohio, United States of America; 4 Corrosion and Protection Center, University of Science and Technology Beijing, Beijing, 100083, P. R. China; LSU Health Sciences Center School of Dentistry, UNITED STATES

## Abstract

Carbon steels are widely used in the oil and gas industry from downhole tubing to transport trunk lines. Microbes form biofilms, some of which cause the so-called microbiologically influenced corrosion (MIC) of carbon steels. MIC by sulfate reducing bacteria (SRB) is often a leading cause in MIC failures. Electrogenic SRB sessile cells harvest extracellular electrons from elemental iron oxidation for energy production in their metabolism. A previous study suggested that electron mediators riboflavin and flavin adenine dinucleotide (FAD) both accelerated the MIC of 304 stainless steel by the *Desulfovibrio vulgaris* biofilm that is a corrosive SRB biofilm. Compared with stainless steels, carbon steels are usually far more prone to SRB attacks because SRB biofilms form much denser biofilms on carbon steel surfaces with a sessile cell density that is two orders of magnitude higher. In this work, C1018 carbon steel coupons were used in tests of MIC by *D*. *vulgaris* with and without an electron mediator. Experimental weight loss and pit depth data conclusively confirmed that both riboflavin and FAD were able to accelerate *D*. *vulgaris* attack against the carbon steel considerably. It has important implications in MIC failure analysis and MIC mitigation in the oil and gas industry.

## Introduction

Microbiologically influenced corrosion (MIC) has become a major problem in the oil and gas industry due to frequent deployment of water flooding in enhanced oil recovery that increasingly leads to water wetting of pipeline walls [1]. Compared with oil wetting, water wetting greatly increase microbial diversity and population. Another key factor is that oil and gas infrastructures are aging allowing more time for microbes to corrode. MIC was regarded as a primary culprit that caused the Alaskan pipeline leak in March 2006, resulting in a major spike in the global oil prices [2]. MIC is also of concern in many other industries such as water utilities and nuclear power plants [3, 4]. Until recently, there has been no clear mechanism that clarifies why and how MIC happens in nature because of its complexity. This makes it difficult to identify the role of MIC in various corrosion failure cases amid a myriad of other factors such as chemical corrosion caused by CO_2_ and H_2_S.

The biocatalytic cathodic sulfate reduction (BCSR) theory proposed by Gu et al. [5] is based on bioenergetics. BCSR explains why and when sulfate reducing bacteria (SRB) attack occurs. The theory states that an SRB biofilm on a steel surface needs energy for its growth or maintenance. When there is a lack of electron donors (e.g., a lack of organic carbon due to diffusional limitation), the sessile cells at the bottom of an SRB biofilm will switch to elemental iron as an alternate electron donor (fuel) for the oxidation of sulfate in its energy production. Opportunistically, these cells may use the elemental iron simply because there are abundantly available nearby. The following equations can be used to explain the bioelectrochemistry in BCSR. In the SRB attack against carbon steel, the anodic reaction is elemental ion oxidation that releases electrons, while the cathodic reaction is sulfate reduction utilizing the electrons.

$$\text{Anodic: Fe}\to {\text{Fe}}^{\text{2+}}+2e^{-}\;(\text{Iron oxidation})$$ (1)

$$E_{e}\text{(V)}=-0.447+\frac{RT}{2F}{\text{ln}[\text{Fe}}^{2+}]\;(\text{vs}.\text{ SHE})$$ (2)

$${\text{Cathodic: SO}}_{\text{4}}{}^{\text{2-}}+{\text{9H}}^{\text{+}}\text{+}8e^{-}\to {\text{HS}}^{-}+{\text{4H}}_{\text{2}}\text{O }(\text{BCSR})$$ (3)

$$E_{e}\text{(V)}=0.252-\frac{2.591RT}{F}pH+\frac{RT}{8F}\text{ln}\frac{\left[{\text{SO}}_{\text{4}}^{\text{2-}}\right]}{\left[{\text{HS}}^{-}\right]}\;(\text{vs}.\text{ SHE})$$ (4)

In the Nernst equations above, R is the universal gas constant, T the absolute temperature and F the Faraday constant. SHE denotes the standard hydrogen electrode. There is actually no physical cathode for the sulfate reduction because it happens in the cytoplasm to SRB cells. The use of the word “Cathodic” here attempts to emulate chemical corrosion mechanisms when both anode and cathode are typically the steel surface. Here the word merely suggests that it is the reduction reaction in the corrosion mechanism.

Unlike an organic carbon, elemental iron in a steel matrix is insoluble. Its oxidation occurs outside SRB cells. The released electrons must be transported across the cell wall into the cytoplasm inside SRB cells because sulfate reduction takes place there intracellularly with enzyme catalysis. This means the SRB biofilm must be electrogenic, i.e., capable of cross-cell wall electron transfer. It utilizes an exogenous oxidant (i.e., sulfate). This type of MIC is classified by Gu [6] and Xu et al. [7] as Type I MIC. It includes other microbes such as nitrate reducing bacteria (NRB) that utilize nitrate as the exogenous oxidant. There is also another major type of MIC known as Type II MIC, which is caused by secreted metabolites that are corrosive oxidants such as protons and organic acids (proton reservoirs). Because the sessile cell density in a biofilm can be 100 times or higher than that of planktonic cells in the bulk fluid, the pH underneath a biofilm, such as a biofilm of an acid producing bacterium (APB), can be much lower than the pH in the bulk fluid. Type II MIC is also electrochemical, involving Reaction Eq (1) as the anodic reaction and Eq (5) as the cathodic reaction.

$$2{\text{H}}^{+}+2e^{-}\to {\text{H}}_{2}$$ (5)

In this case, both anode and cathode are on the steel surface. The oxidation and reductions reactions are no different from the ones in abiotic acid attack such as acetic acid corrosion. It should be noted that Type I and Type II MIC in some cases may occur together. For example, H_2_S is a corrosive metabolite generated by electrogenic SRB in their metabolism. Although in SRB MIC against carbon steel, Type II MIC is much less important than Type I [8].

**Fig 1:**
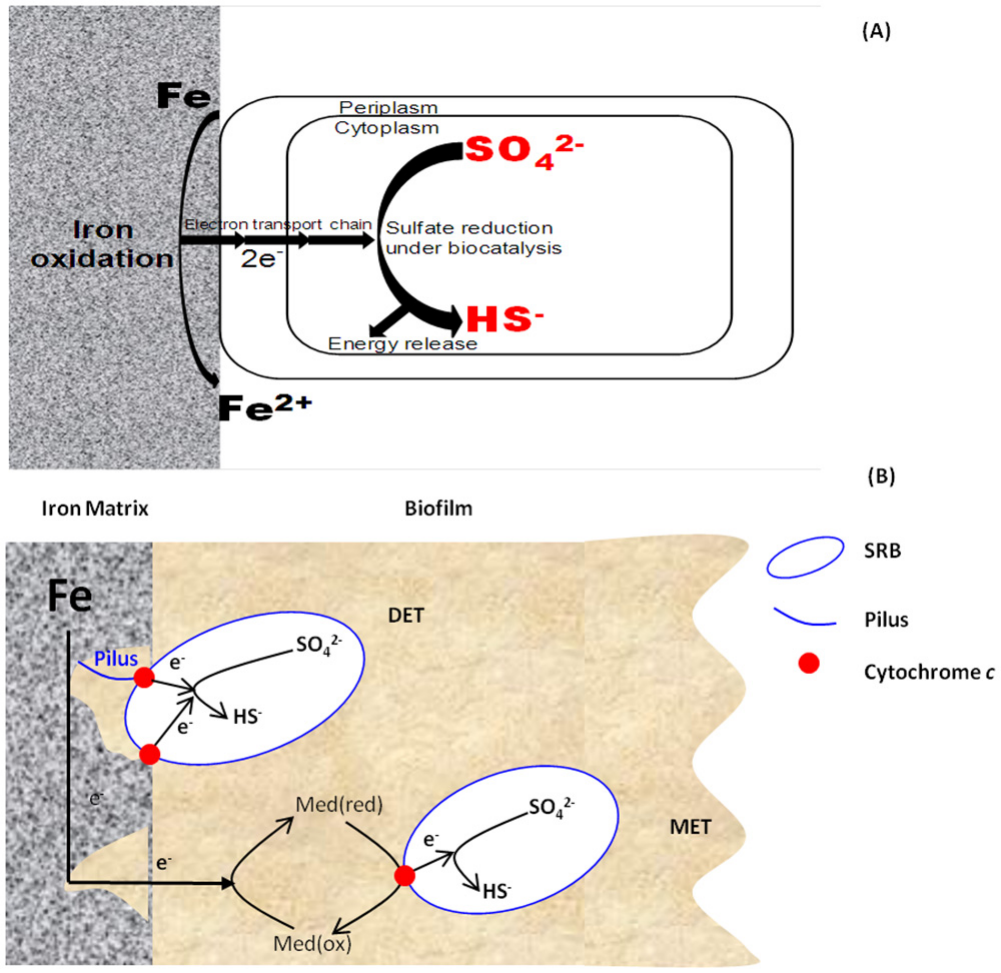
(A) Mechanism for MIC by SRB due to utilization of extracellular electrons from iron oxidation for intracellular sulfate reduction [10], and (B) schematic illustration of DET and MET.

Not all biofilms are electrogenic. When organic carbon is used as the electron donor, cross-cell wall electron transfer (i.e., electrogenicity) is not needed because organic molecules dissolve into the fluid and they are oxidized intracellularly after they diffuse into the cytoplasm. In Type I MIC, electron transfer is likely a limiting step because it is a rather elaborate and difficult process. Hernandez and Newman [9] suggested that extracellular electron transfer (EET) is one of the most fundamental methods for some microbes to generate energy for survival. In fact, EET has been widely investigated in microbial fuel cell (MFC) research in the pursuit of increased electricity output. Du et al. [10] reviewed the two EET types: (a) direct electron transfer (DET), and (b) mediated electron transfer (MET). Both EET types may be used by SRB. Fig 1 illustrates Type I MIC mechanism for SRB and the involvement of DET and MET [11].

In DET, sessile cells attach directly to a steel surface. The membrane-bound *c*-type cytochrome facilitates the EET. For a sessile cell that is very short distance away from a steel surface, conductive nanowires (pili) may be secreted to link a sessile cell with a steel surface for EET. Sherar et al. [12] found that starving SRB secreted pili to link them to a steel surface only when their oil-field SRB was cultured in a culture medium that lacked organic carbon. Apparently, the SRB cells facilitated the harvest of electrons from elemental iron by secreting the pili. Venzlaff et al. [13] also confirmed the direct uptake of electrons from carbon steel by SRB using electrochemical techniques.

**Fig 2:**
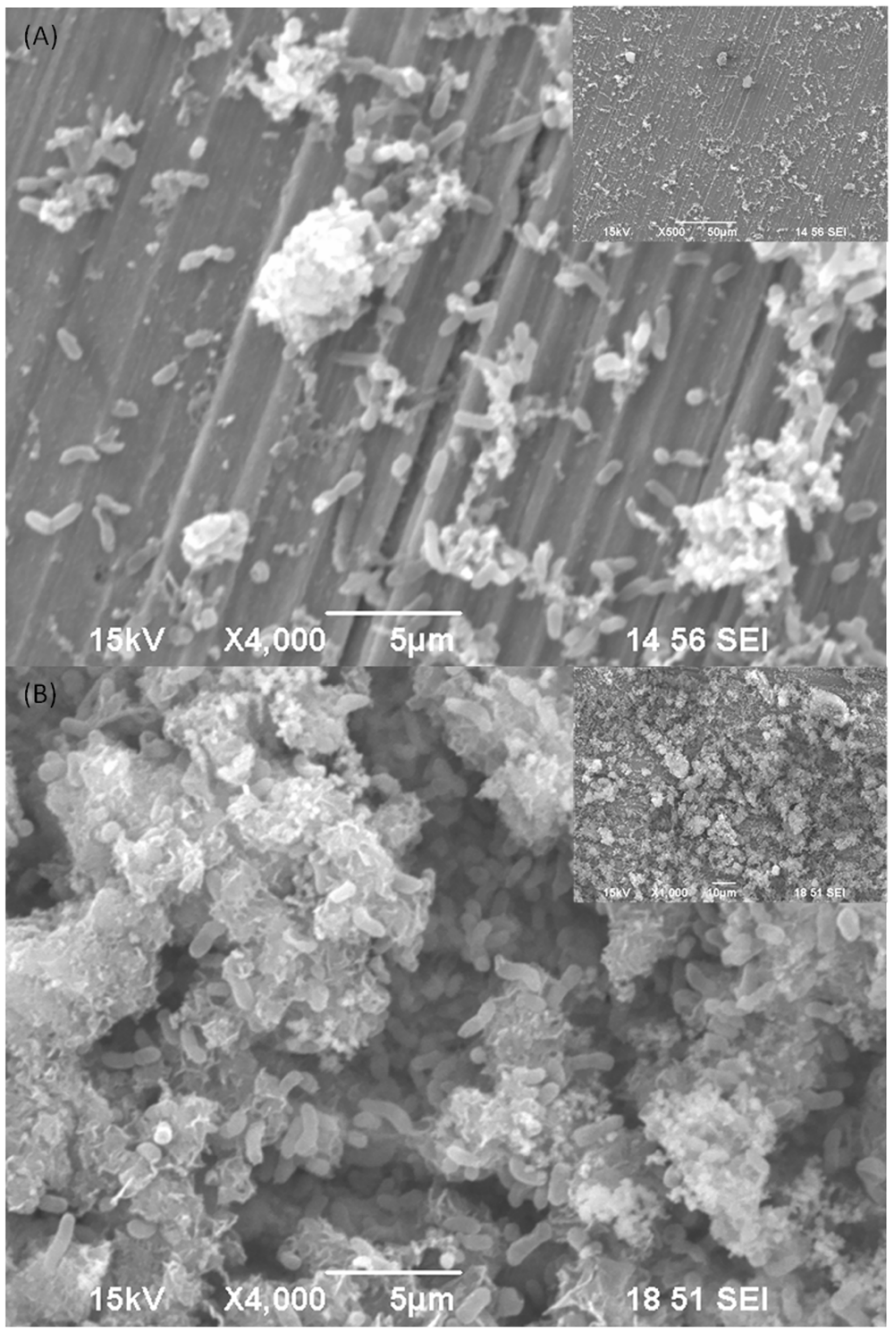
*D*. *vulgaris* biofilms on (A) stainless steel 304 coupon surface and (B) carbon steel C1018 surface after 7 days of incubation with a much higher sessile cell density in ATCC 1249 culture medium.

Zhang et al. [14] demonstrated that two common electron mediators, riboflavin and flavin adenine dinucleotide (FAD), accelerated the MIC of 304 stainless steel by the corrosive SRB *Desulfovibrio vulgaris*. This strongly suggests that EET could be a bottleneck in SRB MIC. In the oil and gas industry, the overwhelming piping material is carbon steels that are used for downhole tubing and transport pipelines. SRB grow much denser biofilms on carbon steels compared with stainless steels as demonstrated by Fig 2. For example, in the absence of any biocide treatment, *D*. *vulgaris* sessile density is 10^4^ times higher on C1018 carbon steel than on 304 stainless steel, leading to much higher weight loss and pit depth for the carbon steel [14, 15]. There have been some field MIC failure cases that showed surprisingly high corrosion rates in carbon steel pipelines that could not be repeated in the lab [16]. Thus, it is imperative to verify the hypothesis that EET is a major bottleneck in Type I SRB MIC attack of a carbon steel, and the MIC can be accelerated considerably in the presence of a naturally occurring electron mediator.

## Materials and Methods

### Bacterium, culture media, chemicals, coupons and MIC testing

Coin-shaped C1018 (UNS G10180) carbon steel coupons with a 1.12 cm^2^ exposed top surface were used. All the other surfaces of the coupon were coated with inert Teflon paint. The coupon preparation procedure followed Xu et al. [17]. The ATCC 1249 medium was used to culture *D*. *vulgaris* (ATCC 7757) at 37°C. The medium components are listed in Table 1. The autoclaved culture medium was sparged with filter-sterilized N_2_ gas for more than 1 h to remove dissolved oxygen before inoculation. Three carbon steel coupons and 100 ml culture medium were placed into each 120 ml anaerobic vial with an initial pH of 7.0 ± 0.2. The initial cell concentration immediately following *D*. *vulgaris* inoculation in each vial was approximately 10^6^ cells/ml. To avoid accidental oxygen ingress, L-cysteine (reagent grade, Fisher Scientific, Pittsburgh, PA, USA) at a concentration of 100 ppm (w/w) was used in the vials as an oxygen scavenger. FAD and riboflavin (both reagent grade, Sigma-Aldrich, St. Louis, MO, USA) were dissolved separately by adjusting the pH of the distilled water. These two electron mediators were each tested at a concentration of 10 ppm (w/w) in the vials. There were three duplicate vials for each test condition. The entire experiment was repeated three times. The manipulations involving the obligate anaerobe *D*. *vulgaris* before the vials were sealed and capped were all performed in an anaerobic chamber filled with filter-sterilized N_2_ gas.

**Table 1:** Composition of ATCC 1249 medium for SRB.

Component I	MgSO_4_	2.0 g
	Sodium Citrate	5.0 g
	CaSO_4_	1.0 g
	NH_4_Cl	1.0 g
	Distilled water	400 ml
Component II	K_2_HPO_4_	0.5 g
	Distilled water	200 ml
Component III	Sodium Lactate	3.5 g
	Yeast Extract	1.0 g
	Distilled water	400 ml
Component IV	Fe(NH_4_)_2_(SO_4_)_2_	Filter-sterilize 5% (w/w) ferrous ammonium sulfate. Add 0.1 ml of this solution to 5.0 ml of medium prior to inoculation.

### Procedures to enumerate SRB and to obtain corrosion weight loss

Planktonic SRB cells in each vial were enumerated on a hemocytometer at 400X magnification every day following Xu et al. [1]. After 7 days of incubation, the coupons were taken out for analysis. The sessile cell counts on the retrieved coupons were enumerated using an SRB test kit (Sani-Check Product #100, Warren, Michigan, USA) following the procedure by Xu et al. [18]. The kit contains a brush dipstick (to remove and collect a biofilm) in a vial filled with a solid SRB medium that turns black when SRB is growing. The time it requires for the black color to appear correlates to the Most Probably Number (MPN) cell counts.

To obtain weight loss, the Clark’s solution (ASTM G1-90 solution for corrosion specimen preparation) was used to remove the biofilm and corrosion products. The coupons were then cleaned with isopropanol and dried in the air. The t-test method was used to analyze corrosion data to obtain the P value for statistical significance.

### Coupon surface analysis

A scanning electron microscope (SEM, Model JSM-6390, JEOL, Japan) was used to examine *D*. *vulgaris* biofilms on coupon surfaces. Before the SEM imaging, coupons were prepared following the procedure described by Xu et al. [7]. To examine the pits underneath biofilms, the coupons were cleaned using Clark’s solution to remove the biofilms and corrosion products on the coupon surfaces. An infinite focus microscopy (IFM) profilometer (Model ALC13, Alicona, Graz, Austria) was used to scan the bare coupon surfaces for pits caused by SRB. The IFM at 5 X magnification was first used to locate the deepest pits on the entire coupon surface. Then, 200 X was used to obtain detailed tomography of the pits.

## Results and Discussion

**Fig 3:**
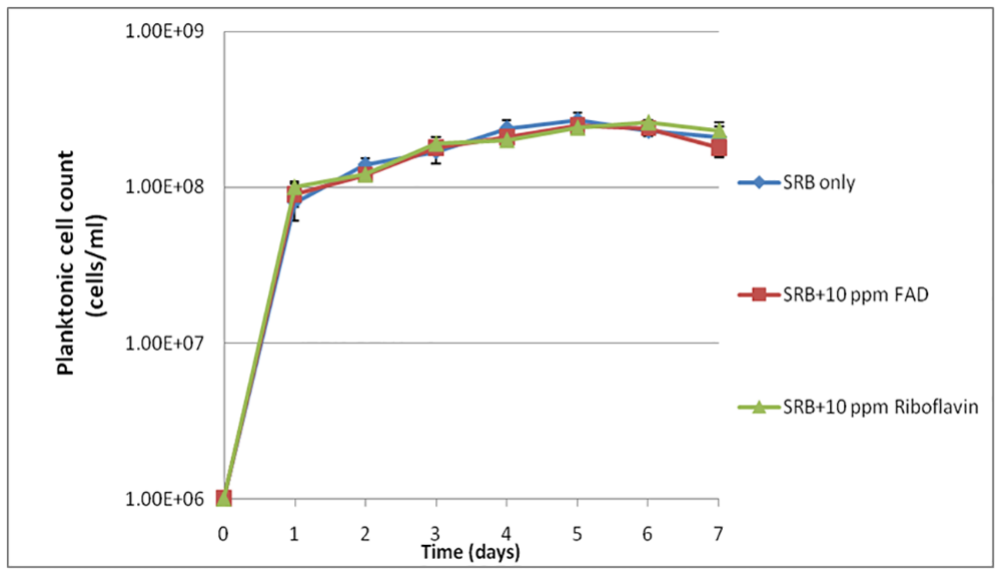
Planktonic *D*. *vulgaris* cell counts after 7 days of incubation with and without electron mediators.

Planktonic SRB cell counts for 7 days are shown in Fig 3. Each data point represents the average reading of three coupons from the same vial. The pH values after 7 days were measured, with the addition of SRB in the absence of a mediator, the pH was 6.6 ± 0.5, while it were 6.7 ± 0.3 and 6.8 ± 0.2, respectively when 10 ppm FAD and riboflavin was added. The results demonstrate that both FAD and riboflavin did not increase the cell concentrations and influence the pH. Lactate in the culture medium was the preferred organic carbon for *D*. *vulgaris* and its concentration (initially 3.5 g/L) was orders of magnitude higher than the concentration (10 mg/L) of the electron mediators. Electron mediators did not show an increase of the sessile cell counts on coupon surfaces either. The MPN sessile cell counts on coupon surfaces were all 10^7^ cells/cm^2^ with and without an added electron mediator. It should be noted that the MPN cell counts are expressed in orders of magnitude because the method could not tell minor differences. The SRB test kit was used to quantify sessile cells because it was difficult to count the sessile cells using a hemocytometer due to presence of large quantities of FeS particles in the sessile cell samples that resembled SRB cells under microscope.

**Fig 4:**
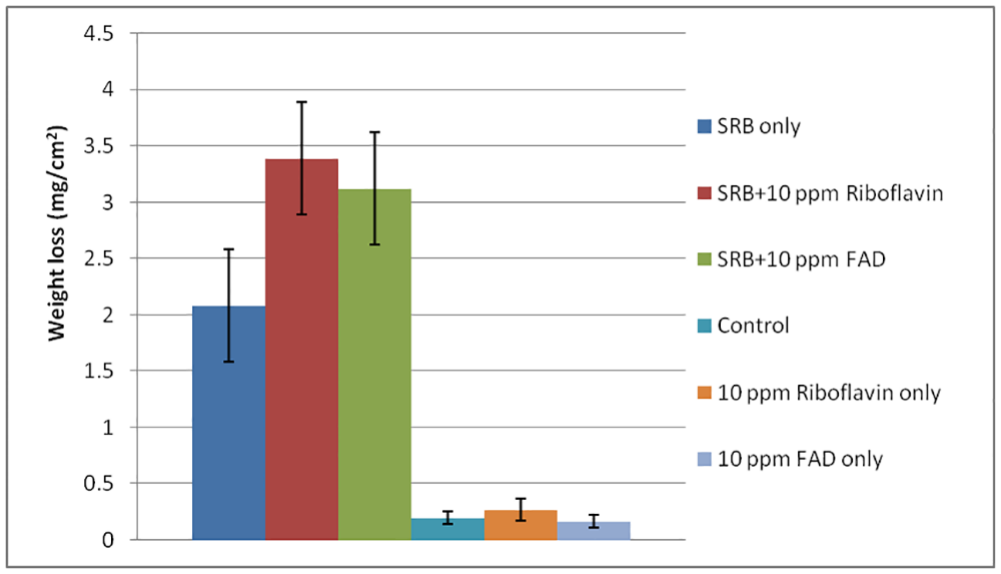
Specific weight loss after 7 days of incubation (error bars representing standard deviations).

**Fig 5:**
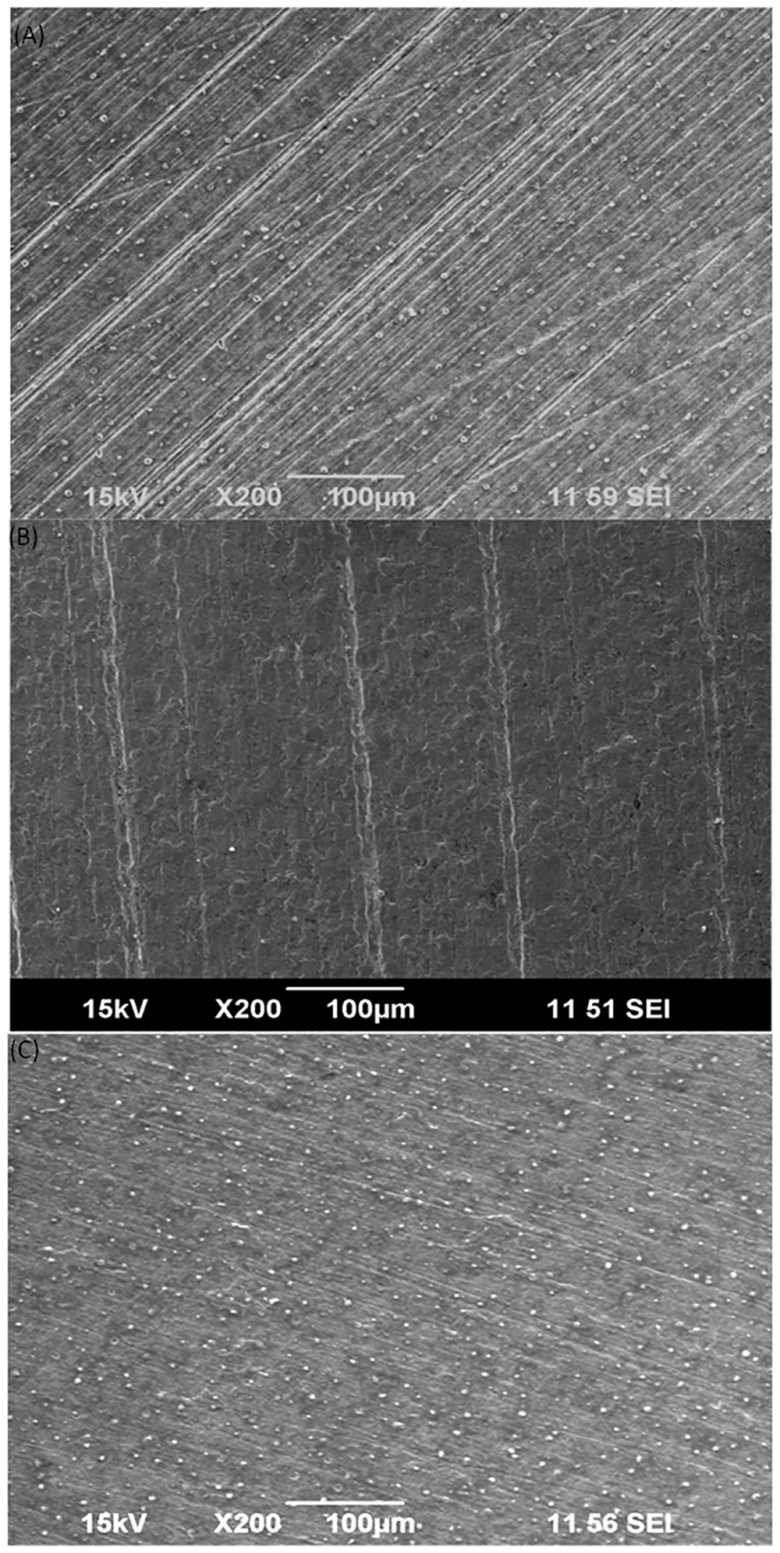
Surface morphology (biofilm removed) under SEM after 7 days of incubation: (A) SRB culture without a mediator, (B) SRB culture with 10 ppm FAD, and (C) SRB culture with 10 ppm riboflavin.

The normalized weight loss data after 7 days in Fig 4 suggest that when an electron mediator was added, the weight loss increased considerably. The average weight loss of the abiotic control was 0.2 mg/cm^2^. With the addition of mediators, the weight loss did not increase suggesting that the mediators themselves were not corrosive. This is consistent with Fig 5, which shows that there were no obvious surface changes on the coupon surfaces when the mediators were added to the abiotic culture medium. With the addition of SRB in the absence of a mediator, the average weight loss was 2.1 ± 0.63 mg/cm^2^, while it reached 3.4 ± 0.70 mg/cm^2^ and 3.1 ± 0.63 g/cm^2^, respectively when 10 ppm FAD and riboflavin was added. They represent a weight loss increase of 62% and 48%, respectively over the control without a mediator. The statistical significance between the SRB culture without a mediator and the addition of a mediator was confirmed by the P values, which were 0.0011 (riboflavin added) and 0.0053 (FAD added), respectively. Both were much smaller than the threshold of 0.05. Thus, the weight loss data clearly indicate that when a mediator was added, the corrosion became more severe.

**Fig 6:**
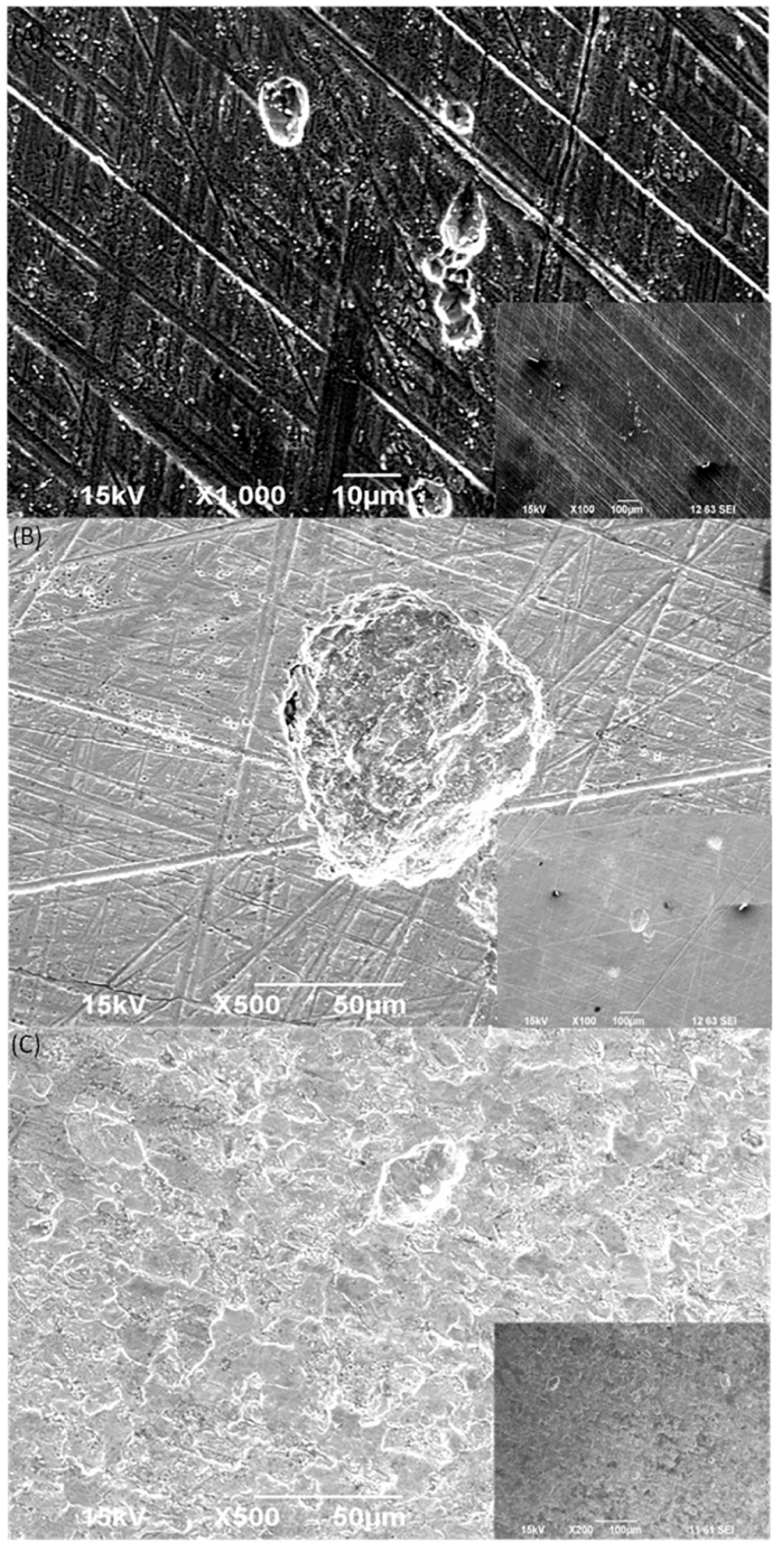
Largest pits in terms of horizontal surface diameter after 7 days of incubation for: (A) SRB culture without a mediator, (B) SRB culture with 10 ppm riboflavin, and (C) SRB culture with 10 ppm FAD.

In Fig 6A, the largest pit caused by the SRB culture without a mediator was approximately 10 μm (horizontal surface diameter). When 10 ppm riboflavin was added, the largest pit shown in Fig 6B was approximately 100 μm, which was 10 times larger than. With the addition of 10 ppm FAD, the largest pit size reached 40 μm (Fig 6C). These pitting data are consistent with the weight loss data above.

**Fig 7:**
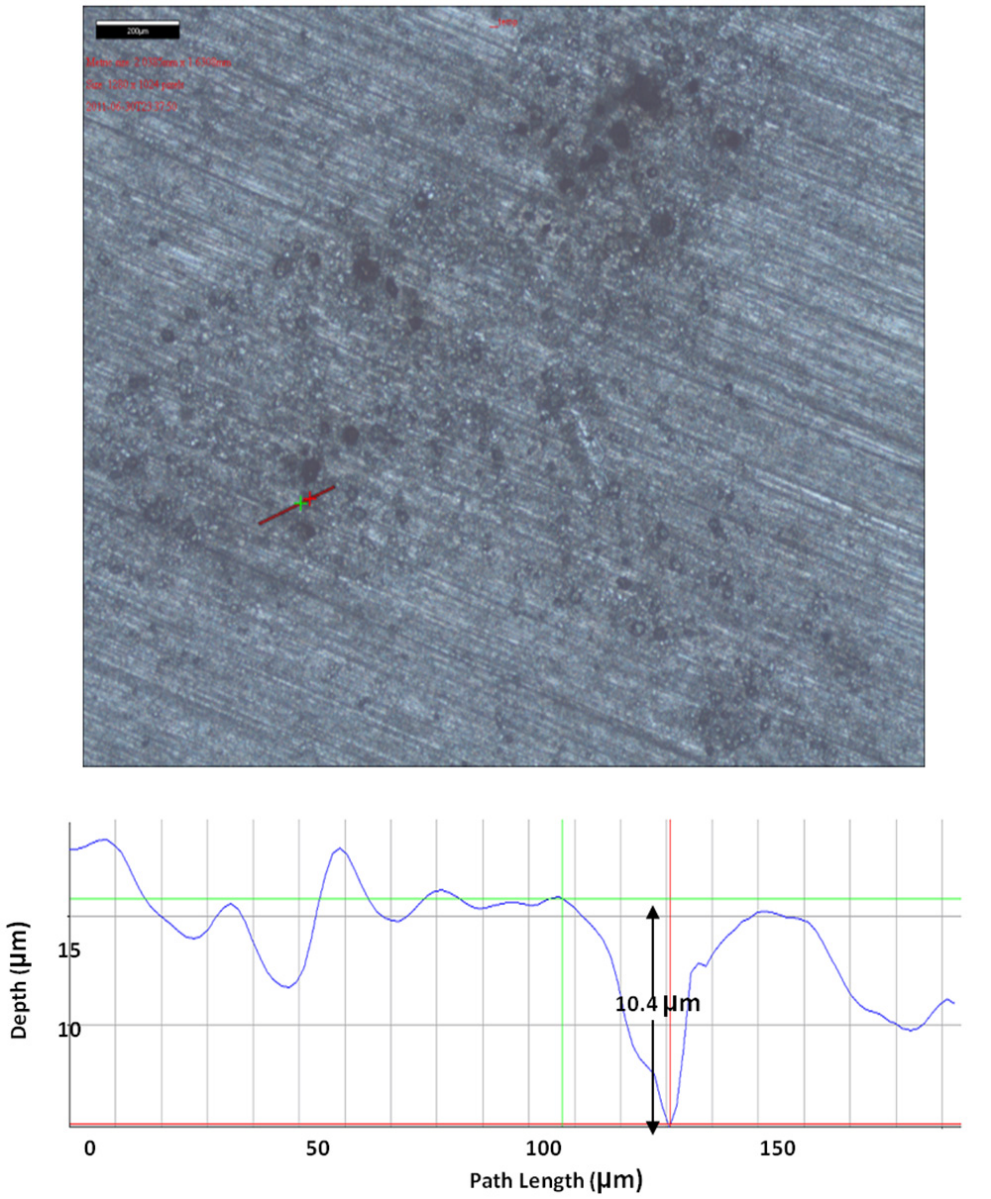
Largest pit depth on a coupon without a mediator after 7 days of incubation was 10.4 μm.

**Fig 8:**
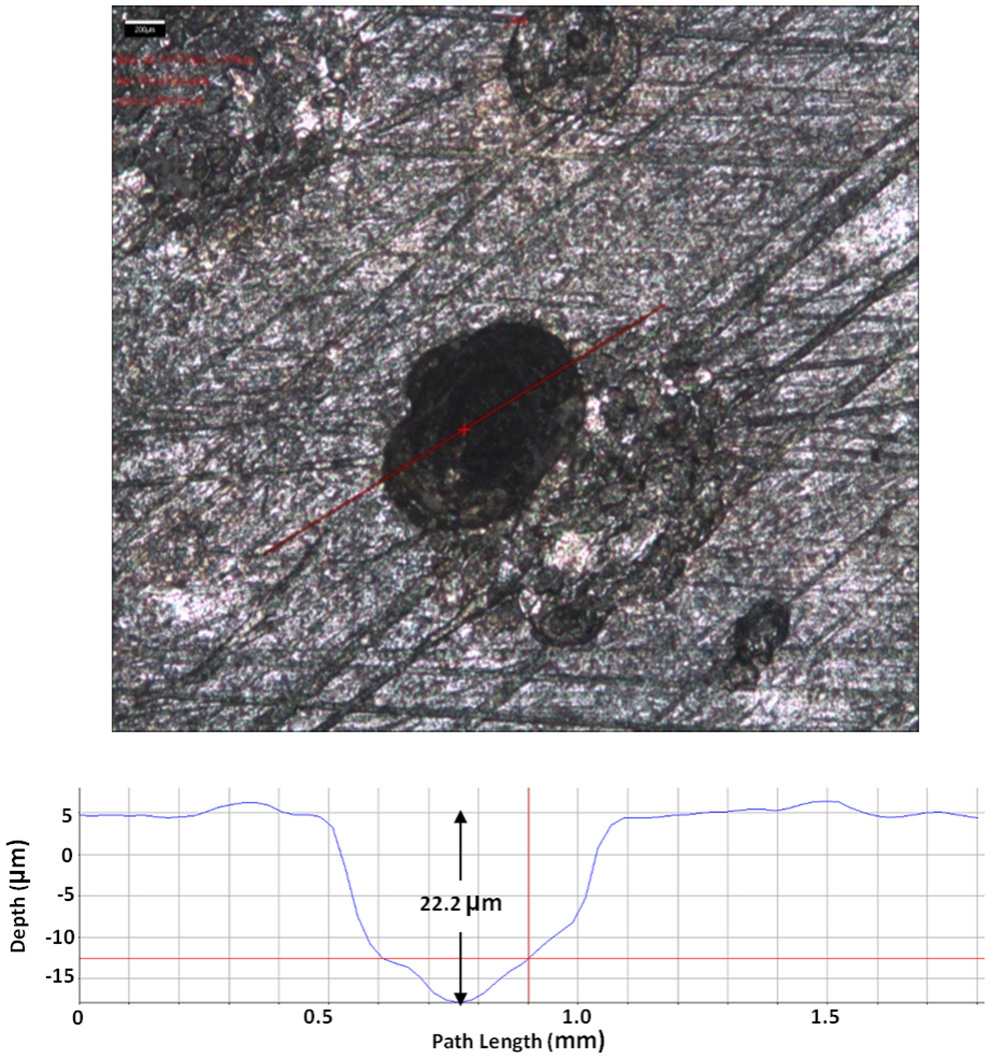
Largest pit depth on a coupon with 10 ppm riboflavin after 7 days of incubation was 22.2 μm.

**Fig 9:**
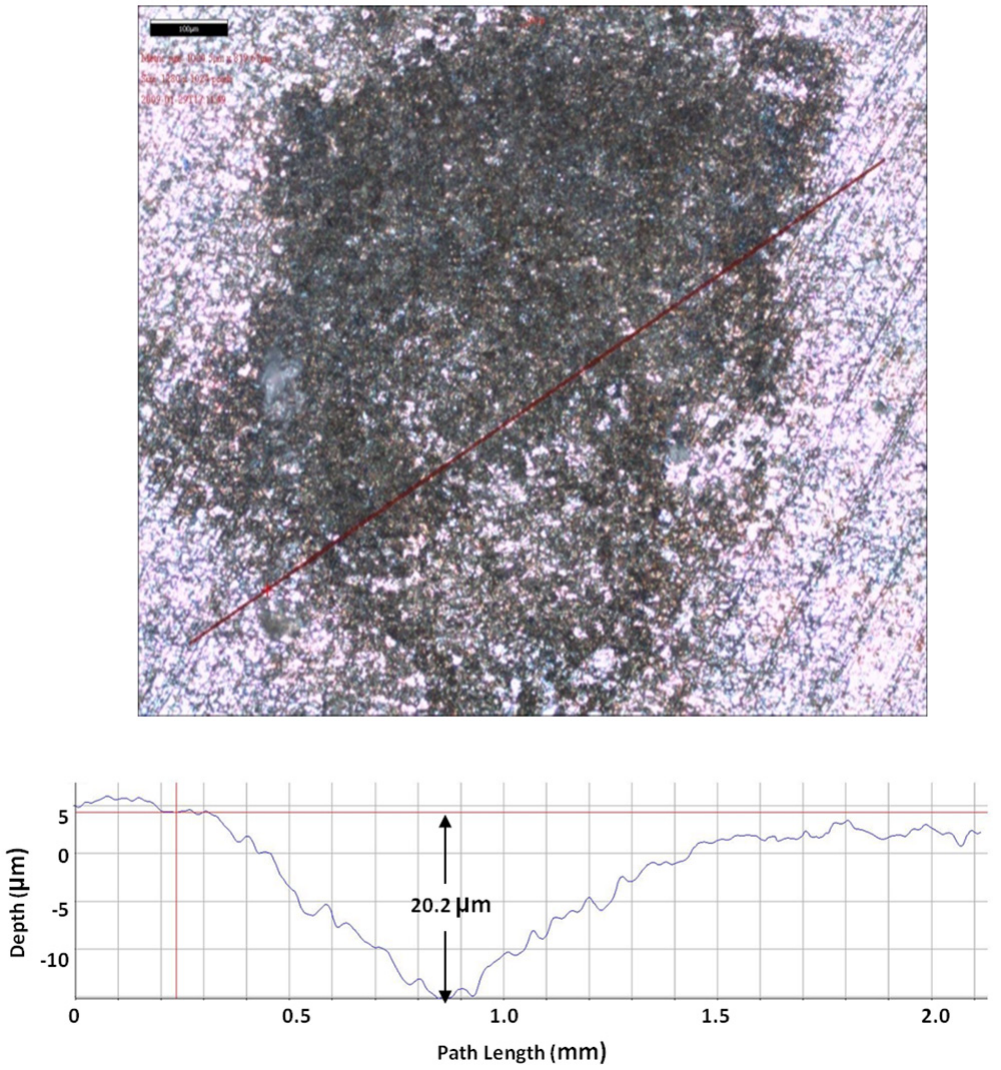
Largest pit depth on a coupon with 10 ppm FAD after 7 days of incubation was 20.2 μm.

Fig 7 shows that the largest pit depth caused by SRB in the absence of a mediator was 10.4 μm. As shown in Figs 8 and 9, with the addition of 10 ppm riboflavin and 10 ppm FAD, the largest pit depth in both treatments was 22.2 and 20.2, respectively. This means that the electron mediators roughly doubled the deepest pit depth compared with the control. MIC failures are typically caused by pinhole leaks. This means the deepest pit matters most. Doubling of the largest pit depth may potentially lead to an MIC pitting failure of a carbon steel pipeline in half of the time.

All the new experimental data above confirm the hypothesis that EET is a key bottleneck in MIC of C1018 carbon steel, and adding an electron mediator considerably increased the MIC by an SRB biofilm. This finding is important to the oil and gas industries, which uses carbon steels for critical installations such as downhole tubing, seawater injection lines, gathering lines and transport lines. Because these environments are typically anaerobic with the presence of sulfate, SRB biofilms are a leading cause of MIC.

In the field, SRB co-exist with other microbes in a synergistic biofilm community. Other microbes in the biofilm consortium may also contribute to the MIC either directly or indirectly. For example, *Shewanella putrefaciens* was found to coexist with SRB in oil pipelines and water tanks [19, 20]. *S*. *putrefaciens* is able to produce extracellular electron mediators such as FAD and riboflavin, which was confirmed in MFC research [21, 22]. In a biofilm consortium, it is possible that *S*. *putrefaciens* (or another microbe) can secrete an electron mediator to facilitate SRB sessile cells’ harvest of electrons from elemental iron. In return, *S*. *putrefaciens* receives energy through the so-called interspecies energy transfer [23]. It has been well known that field MIC corrosion rates can be much higher than those in laboratory tests. This may be due to the synergistic nature of field biofilm consortia that is difficult to reproduce in the laboratory setting.

## Conclusion

Experimental data in this work demonstrated that two common electron mediators, riboflavin and FAD, at a low concentration (10 ppm) both were capable of increasing the MIC of C1018 carbon steel considerably in terms of weight loss and pit size by *D*. *vulgaris*. This work supported the EET aspect of the BCSR theory. It confirmed that MET was an important route for *D*. *vulgaris* electron transfer. The data suggest that cross-cell electron transfer of the electrons released by elemental iron oxidation to the cytoplasm of SRB is a bottleneck in the MIC of C1018 carbon steel by electrogenic SRB. This finding is important in MIC forensics involving unusually fast MIC corrosion rates. There are other practical applications such as deliberately adding electron mediators for accelerated MIC lab testing or for the lab prediction of worst-case scenario (patent pending). It also points out the possibility of suppressing the secretion of electron mediators to reduce MIC.
